# Preliminary Study on the Simulation of a Radiation Damage Analysis of Biodegradable Polymers

**DOI:** 10.3390/ma14226777

**Published:** 2021-11-10

**Authors:** Ha-Eun Shim, Yeong-Heum Yeon, Dae-Hee Lim, You-Ree Nam, Jin-Hyung Park, Nam-Ho Lee, Hui-Jeong Gwon

**Affiliations:** Advanced Radiation Technology Institute, Korea Atomic Energy Research Institute, 29 Geumgu-gil, Jeongeup 56212, Korea; she0805@kaeri.re.kr (H.-E.S.); yhyeon@kaeri.re.kr (Y.-H.Y.); daehee@kaeri.re.kr (D.-H.L.); yrnam@kaeri.re.kr (Y.-R.N.); jhpak@kaeri.re.kr (J.-H.P.); nhlee@kaeri.re.kr (N.-H.L.)

**Keywords:** PLCL, PLDLA, chain scission, degradation, gamma-ray, Geant4, simulation, radiation damage model

## Abstract

In this study, biodegradable poly(L-lactide-co-ε-caprolactone) (PLCL) and poly(L-co-d,l lactide) (PLDLA) were evaluated using Geant4 (G4EmStandardPhysics_option4) for damage simulation, in order to predict the safety of these biodegradable polymers against gamma ray sterilization. In the PLCL damage model, both chain scission and crosslinking reactions appear to occur at a radiation dose in the range 0–200 kGy, but the chain cleavage reaction is expected to be relatively dominant at high irradiation doses above 500 kGy. On the other hand, the PLDLA damage model predicted that the chain cleavage reaction would prevail at the total irradiation dose (25–500 kGy). To verify the simulation results, the physicochemical changes in the irradiated PLCL and PLDLA films were characterized by GPC (gel permeation chromatography), ATR-FTIR (attenuated total reflection Fourier transform infrared), and DSC (difference scanning calorimetry) analyses. The Geant4 simulation curve for the radiation-induced damage to the molecular weight was consistent with the experimentally obtained results. These results imply that the pre-simulation study can be useful for predicting the optimal irradiation dose and ensuring material safety, particularly for implanted biodegradable materials in radiation processing.

## 1. Introduction

Nowdays, biodegradable polymers are widely used as materials for medical devices [1]. Biodegradable polymers, used for decades, include polyesters and their copolymers, such as poly(L-lactic acid) (PLA), poly(ε-caprolactone) (PCL), poly(L-lactide-co-ε-caprolactone) (PLCL), and poly(L-co-d,l lactide) (PLDLA). Among the various materials, PLCL and PLDLA are very valuable materials used in medical applications as implantable devices because of their excellent flexibility and biodegradability [2,3,4]. Sterilization is essential for implantable devices [5], and some established sterilization methods include dry heat, ethylene oxide, steam, and radiation methods [6]. In particular, the gamma or electron beam sterilization process is performed at room temperature and has the advantage of a short sterilization time and low risk of toxic residues [7]. In addition, it has a high sterilization effect for substances that struggle to penetrate into other sterilizing agents [8]. Packaging is used to protect the bioimplantable device from moisture and ions inside the human body [9], the material is sterilized in the entire volume of the product together with the packaging. Because of these advantages, gamma irradiation is the most commonly used method for the sterilization of materials with a high transmittance [10].

However, free radicals generated by radiation energy can propagate within the polymer chain structure and cause a chain reaction, leading to crosslinking [11,12,13]. Therefore, aliphatic polyesters are decomposed in the radiation sterilization process, and the decomposition temperature may change depending on the polymer composition [7,14,15]. In general, the effect of radiation on polymers can cause changes in various properties, such as chemical composition, crystallinity, molecular weight and density, depending on the radiation dose, dose rate, and temperature [6]. In particular, because biodegradable, polymer-based, implantable devices are very important for the performance and service life of materials [16,17], it is necessary to analyze the changes in the properties of polymer materials according to irradiation dose, in preparation for the possibility of material decomposition during the radiation sterilization process. In addition, radiation sterilization is the most suitable and useful material for human insertion among the currently available sterilization methods. Therefore, it is necessary to secure the optimal sterilization dose for each polymer material according to its intended use.

In contrast, irradiation is well known as a very convenient tool for sterilizing biodegradable polymers and transforming polymer materials through crosslinking, grafting, and decomposition [18]. Because crosslinking using radiation may generate radicals within the polymer chain, the functionalization process may be omitted, and the crosslinking reaction may be performed at a low temperature due to its excellent penetrating power. In particular, irradiation with a high-energy radiation such as gamma rays and electron beams was used for the processing and crosslinking of polymers [19,20].

However, radiation accompanies the chain scission reaction and crosslinking simultaneously and may affect basic properties such as reducing the glass transition, crystallization and melting temperature of the polymer. Other physical properties, such as gas permeability, may differ from those of conventional polymers. In particular, because crosslinking generally reduces the degradability of polymers, it can negatively affect the properties of materials designed for degradable devices after the insertion into the human body [16]. Radiation treatment can control the biodegradation time [21]. A compromise must be found between the mechanical properties and the biodegradation time. Therefore, it is expected that the time and cost of material development can be dramatically shortened if the optimal radiation irradiation conditions that are suitable for the required properties can be selected through a pre-simulation study.

In addition, to analyze the damage to polymers caused by radiation, it is necessary to understand radiation physics, dosimetry, chemical analysis, and instrumental analysis applied to the modeling and simulation of the radiation environment [22]. Therefore, it is necessary to investigate the interaction of the polymer of interest with gamma rays. However, because it is difficult to rely solely on repeated testing and dose selection for an accurate analysis [23], some researchers conducted simulation-based correlation studies [24,25,26]. Ghosal et al. performed theoretical simulations to determine the correlation between the morphological changes due to gamma irradiation and other properties such as molecular weight distribution, intrinsic viscosity, and ionic conductivity [24]. Saha et al. conducted a computer simulation study to examine the effects of gamma irradiation on properties such as molecular weight distribution and viscosity [25]. In particular, the number average molecular weight can be an indicator of the critical dose that causes changes in the physical properties of polymers due to radiation [27]. In addition, the quantitative analysis and interpretation of the number average molecular weight is important for understanding the decomposition behavior of polymer materials according to the radiation dose within the simulation.

**Figure 1:**
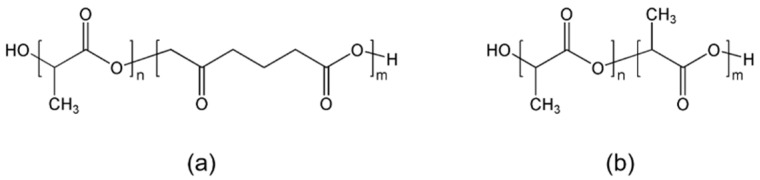
Chemical structure of polymer (**a**) PLCL and (**b**) PLDLA.

In this study, the average molecular weight of the polymer was selected as a parameter to conduct a basic simulation of radiation damage analysis for biodegradable polymers (PLCL and PLDLA). Figure 1 shows the chemical structures of PLCL and PLDLA. In preparation for the possibility of material decomposition during radiation (gamma-ray) sterilization, changes in the characteristics of PLCL and PLDLA according to irradiation dose were observed. In addition, a Geant4 simulation was performed to predict the structural damage dose range in the gamma ray sterilization and processing by selecting this as a damage model, and a comparative verification study was conducted to verify the change in the average molecular weight according to the irradiation dose.

## 2. Materials and Methods

### 2.1. Modeling and Simulation

#### 2.1.1. Geant4 Simulation for Gamma Ray Fluence Calculation

To calculate the radiation dose of polymer films, Geant4 (version 10.6p02, CERN, Conseil Européenne pour la Recherche Nucléaire, Meyrin, Switzerland) was used to perform the simulation. First, the radiation dose of the high-level gamma radiation device was simulated. Using the “GeneralParticleSource (GPS)” and “G4RadioactiveDecayPhysics” modules in Geant4, the ^60^Co decay scheme was modeled. The energy absorbed by an alanine dosimeter (cylindrical type with a radius of 0.24 cm and height of 0.3 cm), placed 15.8 cm from the ^60^Co source, was calculated. The “G4EmStandardPhysics_option4” physics model was used to calculate the absorbed dose of the alanine (material: C_3_H_7_NO_2_, density: 1.42 g/cm^3^) dosimeter when exposed to 1173 keV and 1332 keV gamma rays simultaneously released from the ^60^Co source. The dose amount necessary to calculate the same radiation dose as experimental conditions at the target position was converted to fluence.

#### 2.1.2. Geant4 Simulation for Absorbed Dose Calculation

Simulations were performed to calculate the absorbed dose per unit mass (*D*, eV/g) after irradiating the PLCL and PLDLA films with the previously calculated gamma ray fluence. The polymer film was the same size as the actual film, measuring 2 × 2 × 0.1 cm^3^. The PLCL film was made of HO[C_3_H_4_O_2_]_n_[C_6_H_10_O_2_]_m_CH_3_, (n:m = 70:30), and had a density of 1.2 g/mL. The PLDLA film was made of HO[C_3_H_4_O_2_]_n_[C_3_H_4_O_2_]_m_CH_3_, (n:m = 70:30), and had a density of 1.2 g/mL. The PLCL and PLDLA films were placed 15.8 cm away from the ^60^Co source, and the absorbed dose per unit mass was calculated as summarized in Table 1.

#### 2.1.3. Prediction of Damage to Polymer Materials Using a Radiation Damage Model

The relationship between the radiation dose and number average molecular weight can be derived as follows: The number average molecular weight (*M*_n,0_, g/mole) for the polymer sample is as follows [28]:

*M*_n,0_ (g/mole) = *wN*_A_/*N*_0_ (1)

where *w* denotes the weight (g) of the polymer sample, *N*_A_ is Avogadro’s number, and *N*_0_ is the total number of molecules (initial molecules) in *w* before irradiation. From Equation (1), *N*_0_ can be rearranged as follows:

*N*_0_ = *wN*_A_/*M*_n_ (2)

If the dose is expressed as *D* (eV/g), we can calculate *Dw*, that is, the total absorbed dose by the sample. Therefore, the number of newly formed molecules (N*) inside the polymer due to irradiation is obtained as follows:

N* = *KDw* (3)

where *K* denotes the polymer structure constant that represents the resistance to radiation. It can be replaced by *G*, which is defined as the number of molecules or atoms produced per 100 eV of energy.

The *G*-value is generally expressed as *G*_s_ for the number of scissions due to exposure, or *G*_x_ for the number of crosslinking reactions. The production of new molecules in relation to the number of scissions (*N*_s_*) and crosslinking (*N*_x_*) can be described as follows:

*N*_s_* = (*G*_s_/100) *Dw* (4)

*N*_x_* = (*G*_x_/100) *Dw* (5)

From the perspective of the number of molecules in the polymer sample, chain scissions increase the number of molecules, whereas crosslinking decreases the number of molecules. Therefore, the number average molecular weight (*M*_n_*) of the polymer when the scissions and crosslinking reactions occur competitively with the absorbed dose *D* is obtained as follows:

*M*_n_* = *wN*_A_/(*N*_0_ + *αN*_s_* − *βN*_x_*) (6)

where the total mass of the polymer is assumed to be constant during exposure, *α* and *β* denote constants used to consider the change in the initial number of molecules (*N*_0_) when chain scission and crosslinking events occur; 0.5 and 2 are applied, respectively.

Based on Equations (4)–(6), the polymer damage model that considers radiation-induced chain scission and crosslinking is as follows:

1/*M*_n_* = 1/*M*_n,0_ + [(*αG*_s_ − *βG*_x_)/100*N*_A_] *D* (7)

where *M*_n,0_ and *G*-value (*G*_s_ and *G*_x_) were obtained from the experimental results. The radiation damage model of Equation (7) was used to simulate a biodegradable polymer film.

### 2.2. Film Preparation and Radiation Measurement for Simulation Verification

#### 2.2.1. Materials

Poly(L-lactide-co-ε-caprolactone) (PLCL, 70/30) and poly(L-lactide-co-d,l-Lactide) (PLDLA 70/30) were commercially purchased from RESOMER^®^ (Evonik Health Care Evonik Industries AG, Essen, Germany). Chloroform (CHCl_3_) was selected as a solvent and was supplied by Showa, Tokyo, Japan. All other reagents and solvents were of analytical grade and were used without further purification.

#### 2.2.2. Sample Irradiation

PLCL and PLDLA films were prepared by a solution casting method using CHCl_3_ as a solvent. The PLCL and PLDLA powders were dissolved in CHCl_3_ to obtain 10 wt% and 4 wt% polymer solutions, respectively. The PLCL and PLDLA films were fabricated by pouring a polymer solution into a well-cleaned glass plate and evaporating the solvent in air at room temperature. Dried films were peeled off manually from the glass plate and dried in vacuum oven for 24 h at room temperature. Prior to gamma irradiation, the prepared films were packed with nitrogen gas in glass vials. Gamma irradiation was thereafter performed using a gamma ^60^Co source on the samples with different radiation doses (25, 50, 100, 200, and 500 kGy) at a dose rate of 10 kGy/h. The ^60^Co source (MDS Nordion, Ottawa, Canada, IR 221 n wet storage type C-188) was located at the Korea Atomic Energy Research Institute (KAERI), Jeongeup, Republic of Korea.

#### 2.2.3. Attenuated Total Reflection Fourier Transform Infrared Spectroscopy (ATR-FTIR)

ATR spectra of gamma-ray-irradiated biodegradable polymer films were obtained using an ATR-FTIR spectrophotometer (Bruker TENSOR 37, Bruker Corporation, Billerica, MA, USA). The spectra were measured in the wavenumber range from 500 to 4000 cm^−1^ in ATR mode. Spectra were recorded using Bruker OPUS software (version 8.5, Bruker Corporation, Billerica, MA, USA) at a resolution of 4 cm^−1^.

#### 2.2.4. Thermal Analysis

The thermal properties of the polymer films were tested by difference scanning calorimetry (DSC, Q100, TA Instruments, New Castle, DE, USA). The DSC thermograms of the polymer films were measured from −50 to 250 °C under a nitrogen atmosphere at a heating rate of 5 °C/min, and then were cooled at room temperature. A second heating cycle was then observed by heating the samples from −50 to 250 °C.

#### 2.2.5. Average Molecular Weight

Changes in the molecular weights of the irradiated film were determined by gel permeation chromatography (GPC, PL-GPC 110, Polymer Laboratories, Church Stretton, UK) with the eluent of CHCl_3_ at a flow rate of 1.0 mL/min at 40 °C. The GPC system was equipped with columns of PLgel Guard column 5 μm, PLgel 10 μm Mixed B and PLgel 5 μm 10,000 A (Polymer Laboratories, Church Stretton, UK) calibrated with polystyrene standards.

The changes in molecular weight are related to the radiation chemical yields of crosslinking (*G*_x_) and chain scission (*G*_s_), which determines the extent of chain scission or crosslinking during gamma-ray irradiation, and can be calculated from the following equations [27]:

1/*M*_w_ = 1/*M*_w,0_ + (*G*_s_/2 − 2*G*_x_)*D* × 1.038 × 10^−6^ (8)

1/*M*_n_ = 1/*M*_n,0_ + (*G*_s_ − *G*_x_)*D* × 1.038 × 10^−6^ (9)

where *M*_w,0_ and *M*_n,0_ are the weight and number average molecular weight of unirradiated films. *M*_w_ and *M*_n_ are the corresponding values following exposure to irradiation dose, *D*. A ratio of *G*_s_/*G*_x_ greater than 4 would indicate that chain scission is more prominent for nitrogen atmospheres [29].

## 3. Results and Discussion

### 3.1. Modeling and Simulation

**Figure 2:**
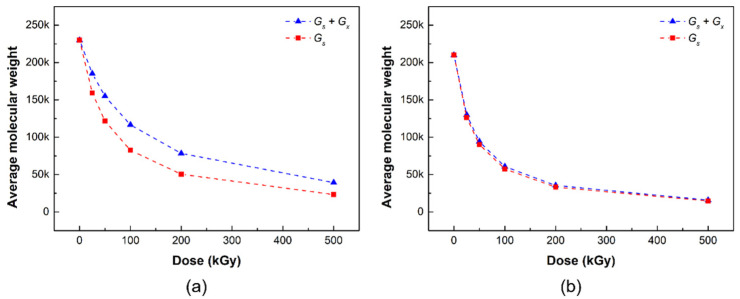
Number average molecular weight of (**a**) PLCL and (**b**) PLDLA radiation damage models depending on the gamma-ray irradiation in simulation (blue triangles: simulation of chain scission and crosslinking; red squares: simulation of chain scission).

Figure 2 shows the number average molecular weight change according to the gamma irradiation dose of the radiation damage model PLCL and PLDLA films through Geant4 simulation. The blue triangle represents the simulation result considering both chain scission and crosslinking in the radiation damage model, whereas the red square represents the simulation result considering only the chain scission in the radiation damage model. The *M*_n,0_ and *G*-values (*G*_s_ and *G*_x_) of PLCL and PLDLA obtained from the experimental results were used in Equation (7) for the radiation damage model of the polymer considering the radiation cleavage and crosslinking derived in this study, respectively.

In the simulation results, the average molecular weight of the radiation damage model PLCL decreased with the increasing irradiation dose (Figure 2a). In the two damage models, the difference in the number average molecular weight in the range 25–200 kGy of the irradiation dose appeared to gradually widen, but the gap seemed to narrow at the irradiation dose of 500 kGy; it appeared that both the chain cleavage and crosslinking reactions occurred up to the range before the irradiation dose of 200 kGy, but the chain cleavage reaction was expected to be relatively dominant at a high irradiation dose of 500 kGy or more.

Figure 2b shows the number average molecular weight changes, according to the gamma radiation dose of the radiation damage model PLDLA film, through the Geant4 simulation. In the simulation results, the number average molecular weight of the radiation damage model PLDLA decreased with the radiation dose. Interestingly, when only chain cleavage was considered in the radiation damage model, there was no significant difference from when both chain cleavage and crosslinking were considered, that is, in the PLDLA damage model, it can be inferred that the chain cleavage reaction prevails over crosslinking.

### 3.2. Radiation Assessment for Simulation Verification

#### 3.2.1. ATR-FTIR Spectroscopy

**Figure 3:**
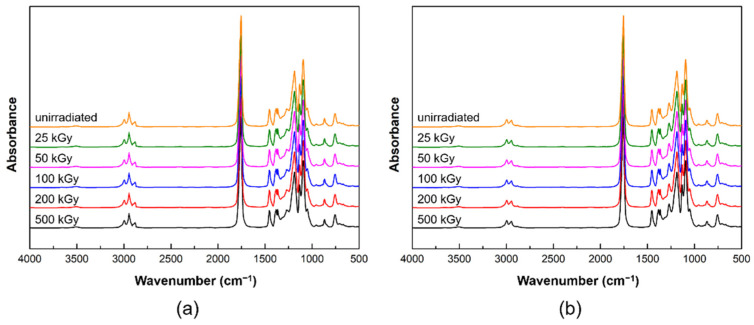
ATR-FTIR spectra before and after gamma-ray irradiation: (**a**) PLCL and (**b**) PLDLA polymer films.

Figure 3 shows the ATR-FTIR spectra of PLCL and PLDLA before and after gamma-ray irradiation. The ATR-FTIR spectrum of the PLCL film was observed from 1300 to 1000 cm^−1^, which is related to the stretching of C−O bonds in the ester, found in the long alkyl chain of the polymer structure [30,31]. The C−O and C−O−C groups exhibited stretching peaks at 1188, 1037, and 1079 cm^−1^ for the PLCL film both before and after exposure. The stretching of −C=O (carbonyl) appeared as an intense peak at 1750 cm^−1^. In addition, the asymmetric stretching vibration of the −CH_3_ and −CH_2_ groups were observed at 2992 and 2943 cm^−1^ for the PLCL film before and after exposure [29,31,32]. Another bond related to the symmetric vibration of −CH_2_ was observed at 2872 cm^−1^ [29]. Bonds associated with the asymmetrical and symmetrical stretching of the −CH_3_ group exhibited peaks at 1450 and 1361 cm^−1^, respectively [33]. Small peaks were also observed for the C−H bending vibrations at 757 and 864 cm^−1^ [34].

Figure 3b shows the ATR-FTIR spectra of PLDLA before and after gamma-ray irradiation. The C−O and C−O−C groups exhibited stretching peaks at 1182 and 1081 cm^−1^ for the PLDLA film both before and after exposure. The stretching of −C=O (carbonyl) appeared as an intense peak at 1746 cm^−1^. In addition, the asymmetric stretching of −CH was observed at 2994 and 2945 cm^−1^ for the PLDLA film before and after exposure. Another bond related to the bending vibration of −CH_3_ was observed at 1452 cm^−1^ [33]. Small peaks were also observed for the C−H bending vibrations at 759 and 872 cm^−1^ [34].

The PLCL and PLDLA films did not display significant differences before and after exposure due to minor radiation-induced chemical changes occurring in the polymer chain [35,36]. This implies that there was no change in the functional group inside the polymer after irradiation, and this result indicated that the possibility of the creation of a new bond, which was one of the parts to be considered in the decomposition mechanism of the polymer, could be excluded.

#### 3.2.2. Thermal Analysis

**Figure 4:**
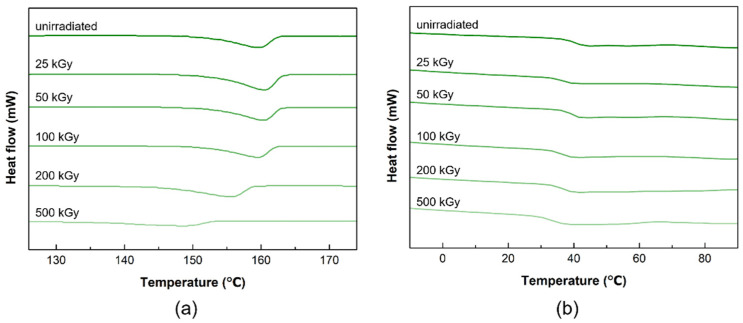
DSC thermograms of the before and after gamma-ray irradiation (**a**) PLCL and (**b**) PLDLA polymer films.

Figure 4 shows the DSC curves of PLCL and PLDLA before and after gamma-ray irradiation. The DSC curve of the PLCL film before exposure had a melting point (*T*_m_) of 159.78 °C. No significant differences were observed up to a dose of 100 kGy. However, *T*_m_ decreased rapidly when the dose was increased to 200 and 500 kGy, indicating that gamma-ray irradiation resulted in the degradation and scissioning of the main chain [20,37].

In the DSC curve of the PLDLA film before irradiation, a glass transition (*T*_g_) was observed at 41.75 °C. After gamma irradiation, there was no significant change at 100 kGy or less, and a decrease in *T*_g_ was observed at 200 kGy. The *T*_g_ of a polymer was related to its molecular weight, and *T*_g_ decreases as the average molecular weight decreases [38]. Consequently, it was confirmed that the temperature at which thermal transition occurs changed when the dose was greater than 100 kGy.

#### 3.2.3. Average Molecular Weight

**Figure 5:**
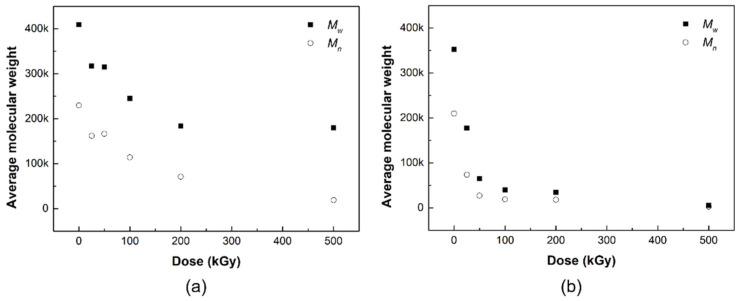
Average molecular weight (black squares: *M*_w_, white circles: *M*_n_) of (**a**) PLCL and (**b**) PLDLA depending on the gamma-ray irradiation.

The changes in the average molecular weights (*M*_w_ and *M*_n_) of the PLCL and PLDLA films before and after gamma-ray irradiation are shown in Figure 5. The oxidation of polymers during exposure reduces crosslinking, increases degradation, or causes chain scissions [24]. Thus, the formation of radicals after exposure results in chain scissions, which in turn lowers the molecular weight. Chain scissions usually occur when polymers are in the amorphous phase [25].

At higher doses of at least 200 kGy, chain scission occurs because of the alkyl free radicals that react with oxygen to form peroxyl free radicals through hydrogen abstraction [26,27]. This type of chain scission has no significant effect on the decrease in the average molecular weight, given the relatively higher increase in chain scission, compared to crosslinking events under higher doses [39]. The number of alkyl free radicals was greater than that of peroxyl free radicals under higher radiation doses because oxygen was not present under our experimental conditions. Alkyl free radicals had less influence on chain scissions than peroxyl free radicals, and were more likely to undergo rebonding or crosslinking in crystalline and amorphous segments [40].

Hydrogen abstraction due to chain-breaking radicals in the weakest C–H bonds contributes to an increase in alkyl radicals or –C(CH_3_)– radicals. The average molecular weight decreased with the degradation of the polymer during exposure [41]. From Table 2, the high *G*-value (*G*_s_/*G*_x_ = 8.7) exhibits a degree of chain scission in the irradiated PLCL film after exposure. The decrease in molecular weight at low radiation doses was due to chain scission by alkyl free radicals [26]. High-energy irradiation forms radicals played a role in the degradation of polymers [41]. In other words, the chain appears to be affected at a dose of 30 kGy or higher, but exhibits a more distinct difference in molecular weight at higher doses of at least 200 kGy. Even in the absence of oxygen in the air, radicals that cause chain cleavage may be formed by radiation energy, which may affect the average molecular weight of the polymer.

Polymers with oxygen atoms are known to exhibit a very high sensitivity to radiation [2], and similar studies were conducted on structures containing oxygen in the polymer backbone [29]. Methine groups in the polymer backbone appear to be important in the radiolysis of biodegradable polymers containing oxygen atoms. Previously, Nugroho et al. reported a study on the gamma-ray degradation of PLA [42]. This polymer contains an ester linkage and a cleavage site in the methine group. The cleavage of the ester bond at this cleavage site causes crosslinking with a relatively low yield, whereas cleavage at the methine group causes the chain scission of the polymer [17,42].

In general, the radicals generated at the ends of polymer chains generate new radicals, move to adjacent polymer chains, or cause hydrogen abstraction [42]. In addition, double bonds are formed at the chain ends after hydrogen abstraction. In PLDLA, a copolymer comprising of isomers of PLA, and both crosslinking reactions, can cause decomposition reactions due to cleavage of ester bonds in the polymer and hydrogen abstraction of the methine groups [20]. In this study, when interpreted only as a result of the decrease in the average molecular weight of PLDLA according to gamma radiation dose, the radicals generated inside the polymer react well with each other and the decomposition reaction is relatively dominant; therefore, it is believed that primarily chain cleavage occurs. Consequently, it is observed that the polymer chain is affected by radiation even at a low gamma irradiation of 25 kGy or higher, but it can be confirmed that a distinct change is exhibited with a molecular weight at 50 kGy or higher.

#### 3.2.4. Modeling and Simulation Verification

**Figure 6:**
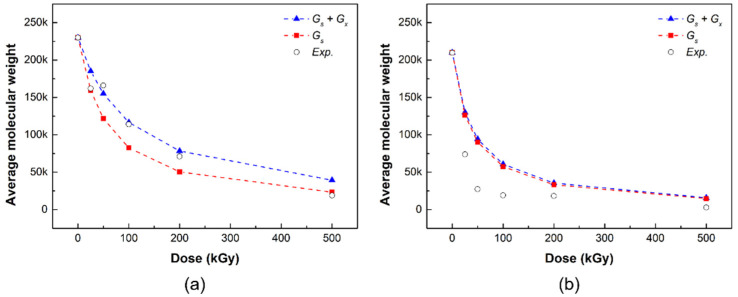
Comparison of number average molecular weight of (**a**) PLCL and (**b**) PLDLA in simulation and measurement evaluation (blue triangles: simulation of chain scission and crosslinking, red squares: simulation of chain scission, white circles: experimental value).

Figure 6 shows the results of the simulation of the radiation damage model and the change in the number average molecular weight of the PLCL and PLDLA films according to the gamma radiation dose. The blue triangle represents the simulation result considering both chain scission and crosslinking in the radiation damage model, the red square represents the simulation result considering only the chain scission in the radiation damage model, and the white circle represents the experimental result obtained from the radiation measurement evaluation.

The simulation results predicted that, in the radiation damage model, both chain cleavage and crosslinking reactions occurred at low doses before 200 kGy, but chain cleavage reactions were relatively dominant at high doses of 500 kGy or higher (Figure 6a). The number average molecular weight of the PLCL film decreased according to the gamma radiation dose in the radiation measurement results, which exhibited a similar trend to the Geant4 simulation results. The actual evaluation results were similar to those of the radiation damage model when both chain scission and crosslinking were considered at a radiation dose in the range 0–200 kGy. Meanwhile, the actual evaluation results at a dose of 500 kGy were similar to the simulation results of the radiation damage model considering only chain scission. Consequently, it can be determined that PLCL has a more dominant chain cleavage reaction than crosslinking at a high radiation dose of at least 500 kGy, and the simulation of the radiation damage model can be verified through a comparison with the experimental results.

The simulation results of PLDLA exhibited no significant difference when only chain cleavage was considered in the radiation damage model, compared to when both chain cleavage and crosslinking were considered (Figure 6b). Therefore, the PLDLA damage model predicted that the chain cleavage reaction would prevail at the total irradiation dose (25–500 kGy). The results of the radiation measurement demonstrated that the polymer chain was affected by radiation even at a low irradiation dose of 25 kGy or higher, and that the chain cleavage reaction was dominant, with a distinct molecular weight change at 50 kGy or higher. Consequently, unlike PLCL in which cleavage was predominant at high doses, in the case of PLDLA, it could be determined that the chain cleavage reaction was dominant at the total irradiation dose (25–500 kGy), and the simulation could be verified through a comparison with experimental results.

In addition, in Equation (7) derived from this study, the slopes *α* and *β* are correlated with the G value, and thus the radiation damage model is an important factor in simulating the actual evaluation experiment. To reflect the change in the initial number of molecules (*N*_0_) due to molecular formation through chain cleavage and crosslinking, 0.5 and 2 were applied to the slopes *α* and *β*, respectively. The average molecular weight change curve was also determined.

Regarding the degradation of biodegradable polymers, as well as the results of our study, the results of modeling and simulation studies by other researchers were also reported [43,44,45]. This work focused on the average molecular weight required to conduct a basic simulation of radiation damage analysis for biodegradable polymers. However, the analysis reported in this paper corresponds to a simplified model of the interaction of ionizing radiation through only single ionization. It was not fully considered that, as a result of radiolysis, apart from molecular hydrogen, molecular oxygen was also released [46]. Thus, it is necessary to carry out additional research considering post-radiation, oxidative degradation processes.

## 4. Conclusions

In this study, PLCL and PLDLA, which are representative, implantable polymer materials, were selected as target material models to conduct a predictive simulation study on the radiation damage and prediction of biodegradable polymer materials.

A decomposition evaluation using gamma rays was performed through the characterization (ATR-FTIR, GPC, and DSC thermal analysis) of PLCL and PLDLA films according to the irradiation dose. No clear structural decomposition was observed in the ATR-FTIR results, but changes in the melting point (*T*_m_) and average molecular weight (*M*_n_ and *M*_w_) of the PLCL film decreased as the irradiation dose increased, as shown by the DSC and GPC analysis results. Similarly, in the PLDLA film, no clear structural decomposition was observed in the ATR-FTIR results. Yet, in the GPC analysis results, a decrease in the average molecular weight (*M*_n_ and *M*_w_) of the PLDLA film was observed as the irradiation dose increased. In contrast, a change in the glass transition temperature (*T*_g_) was observed at irradiation doses greater than 100 kGy, and it seemed that the optimal sterilization dose to maintain thermal stability could be derived.

In addition, PLCL and PLDLA films were selected as damage analysis and prediction models and were manufactured in the form of films; a Geant4 simulation was performed using gamma rays. Consequently, a number average molecular weight (*M*_n_) change curve similar to the actual measurement result was confirmed. This study is a preliminary study for the analysis and prediction of radiation damage of biodegradable polymer materials using gamma rays, which could be possible.

## Figures and Tables

**Table 1:** Absorbed dose per unit mass of PLCL and PLDLA models according to gamma radiation dose.

Dose (kGy)	PLCL	PLDLA
	Absorbed Dose per Unit Mass (eV/g)	
25	1.47 × 10^21^	2.73 × 10^20^
50	2.95 × 10^21^	5.47 × 10^20^
100	5.90 × 10^21^	1.09 × 10^21^
200	1.18 × 10^22^	2.19 × 10^21^
500	2.95 × 10^22^	5.47 × 10^21^

**Table 2:** Chain scission (*G*_s_) and crosslinking (*G*_x_) radiation yields of PLCL and PLDLA irradiated.

Polymer (dose)	*G*_s_	*G*_x_	*G*_s_/*G*_x_
PLCL (200)	0.158	0.018	8.7
PLDLA (100)	1.394	0.026	52.7

## Data Availability

Not applicable.
